# Seasonal variation of non-shivering thermogenesis (NST) during mild cold exposure

**DOI:** 10.1186/s40101-015-0051-9

**Published:** 2015-03-13

**Authors:** Takayuki Nishimura, Midori Motoi, Yuka Egashira, Damee Choi, Kiyoshi Aoyagi, Shigeki Watanuki

**Affiliations:** Department of Public Health, Nagasaki University Graduate School of Biomedical Sciences, 1-12-4 Sakamoto, Nagasaki, 852-8523 Japan; Department of Kansei Science, Graduate School of Integrated Frontier Sciences, Kyushu University, 4-9-1 Shiobaru, Minami-ku, Fukuoka 815-8540 Japan; Department of Human Science, Faculty of Design, Kyushu University, 4-9-1 Shiobaru, Minami-ku, Fukuoka 815-8540 Japan

**Keywords:** Non-shivering thermogenesis, Oxygen intake, Cold exposure, Seasonal acclimatization

## Abstract

**Background:**

The physiological function of non-shivering thermogenesis (NST) has been investigated in recent years, and some studies have discussed the importance of NST with respect to human cold adaptation. The present study aimed to clarify individual and seasonal variations in NST that occurred as a result of mild cold exposure.

**Methods:**

Seventeen male university students participated in the present study during summer and winter. The climate chamber used was programmed so that ambient temperature dropped from 28°C to 16°C over an 80-min period. Physiological parameters of test subjects were recorded during the experiments.

**Results:**

Increases in oxygen intake (VO_2_) during cold exposure were significantly greater without shivering in winter than they were in summer. Respiratory exchange ratio (RER) was significantly lower during thermoneutral baseline and cold exposure in winter than it was during the same periods in summer. In addition, there was a significant negative correlation between ΔVO_2_ and ΔRER.

**Conclusions:**

Increase of VO_2_ without shivering indicated increase of NST, and decrease of RER depends on the metabolization of fat in winter. These results suggested that NST activity was activated by seasonal acclimatization, and individual variation of NST depends on individual variation of fat metabolism.

## Background

Adaptation to cold environments played an important role in the survival of *Homo sapiens* during the last ice age, and variations with respect to cold adaptation are reflected in human phenotypes today [1,2]. When humans are exposed to cold environments, vasoconstriction occurs to regulate heat loss; however, the degree to which the thermal environment can be adjusted by vasoconstriction is small, and thermogenesis is required to maintain optimal body temperature.

Thermogenesis can be divided into shivering thermogenesis (ST) and non-shivering thermogenesis (NST); the former is considered to be the main form of thermogenesis in humans. In laboratory studies, we previously demonstrated seasonal variation in the lower respiratory exchange ratio (RER) with shivering during acute cold exposure (10°C) in winter [3]. RER is defined as the ratio of carbon dioxide output (VCO_2_) to oxygen intake (VO_2_). High RER values indicate glucose metabolism, while low RER values indicate fat metabolism. Mäkinen *et al*. [4] reported that some individuals exhibited increased VO_2_ without shivering during a 24-h period of cold exposure (10°C) in winter. In addition, Vybúral [5] reported the importance of hormonal effects on NST in winter swimmers. These results suggested that seasonal acclimatization of thermogenesis occurred by including NST.

To better understand energy expenditure during cold exposure, it is necessary to examine ST and NST separately and to elucidate seasonal variation in NST. The present study aimed to elucidate seasonal variation of NST through mild cold exposure. It was hypothesized that energy expenditure would increase without shivering in winter.

## Methods

### Participants

Participants in the study comprised 17 university students (20 to 24 years old) with no known medical problems. All were Japanese men and were non-athletes. After having the experimental conditions fully explained to them, participants gave written consent to their participation. Table 1 shows the morphological characteristics of the participants during each season. Body mass index (BMI) was calculated as follows: body mass (kg)/height^2^ (m^2^). Body surface area (BSA) was calculated using Kurazumi’s formula [6]. Body fat was calculated by Brozek’s formula [7].

**Table 1:** **Participants’ morphological characteristics**

	**Height (cm)**	**Body mass (kg)**	**Body fat (%)**	**BMI**	**BSA (m** ^**2**^ **)**
Summer	172.5 ± 7.7	59.3 ± 7.2	13.1 ± 2.7	19.9 ± 2.0	1.70 ± 0.12
Winter	172.6 ± 7.5	58.9 ± 6.9	12.6 ± 1.8	19.8 ± 1.9	1.70 ± 0.11

No significant differences between the summer and winter values of participants’ morphological characteristics were observed. All data are shown as mean ± S.E. BMI, body mass index; BSA, body surface area.

Experiments were approved by the Ethics Committee of the Graduate School of Design, Kyushu University.

### Experimental procedure

**Figure 1:**
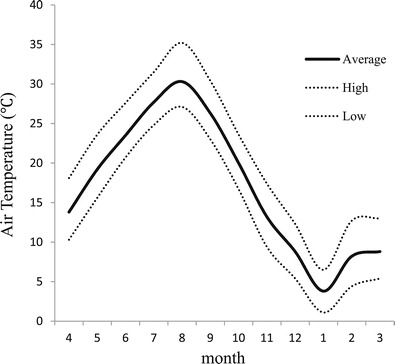
**Changes in average air temperature.** The solid line indicates average air temperature, and the dotted line indicates average high and low temperatures. Data source provided by the Japan Meteorological Agency.

Experiments were conducted in summer (August to September) and winter (February to March) in Fukuoka, Japan. Average temperature during experiment in Fukuoka was 28.3°C in summer and 8.5°C in winter (Figure 1) (Japan Meteorological Agency). Participants abstained from food and drink for at least 2 h prior to experimentation.

Prior to experimentation, sensors were attached to each participant at an ambient temperature of 28°C. Participants then rested quietly for a period of 20 min in a climate chamber prior to commencement of cold exposure. The climate chamber used was programmed to gradually decrease the ambient temperature from 28°C to 16°C over approximately 80 min.

Rectal temperature probes were inserted to a depth of 13 cm beyond the anal sphincter. Skin temperature sensors were attached with surgical tape to measurement sites on the forehead, abdomen, forearm, hand, thigh, leg, and foot. Measurements were made at intervals of 2 s using a data logger (LT-8A, Gram Corporation, Saitama, Japan). Mean skin temperature was calculated using the seven-point method of Hardy-DuBois [8]. VO_2_ and VCO_2_ were measured using a respiratory gas analyzer (AE-300S, Minato Medical Science, Osaka, Japan) in conjunction with a breathing tube, with a Rudolph mask used to measure expired gas (Rudolph mask, Nihon Kohden, Tokyo, Japan). To facilitate comparison with our previous studies and other studies, VO_2_ was divided by body mass, not fat-free mass. Electromyograms of the *pectoralis major* muscle were recorded by electromyograph (PolyTele, Nihon Santeku, Kyoto, Japan). Electromyogram data were recorded at a sampling frequency of 1,000 Hz, and a bandpass filter (20 to 500 Hz) was used in the analysis. Electromyographic data obtained during cold exposure were based on muscular changes during the first 10 min of thermoneutral baseline in 28°C.

### Statistical analysis

Morphological data were compared by the paired *t* test. Physiological data were compared using two-way, repeated-measures, analysis of variance (ANOVA) (season and time), and Ryan’s method was used for *post hoc* tests. The Pearson product-moment correlation analysis was used to determine the relation of ΔRER to ΔVO_2_. All data were expressed as the mean ± standard error (*P* < 0.05).

## Results

### Oxygen intake

**Figure 2:**
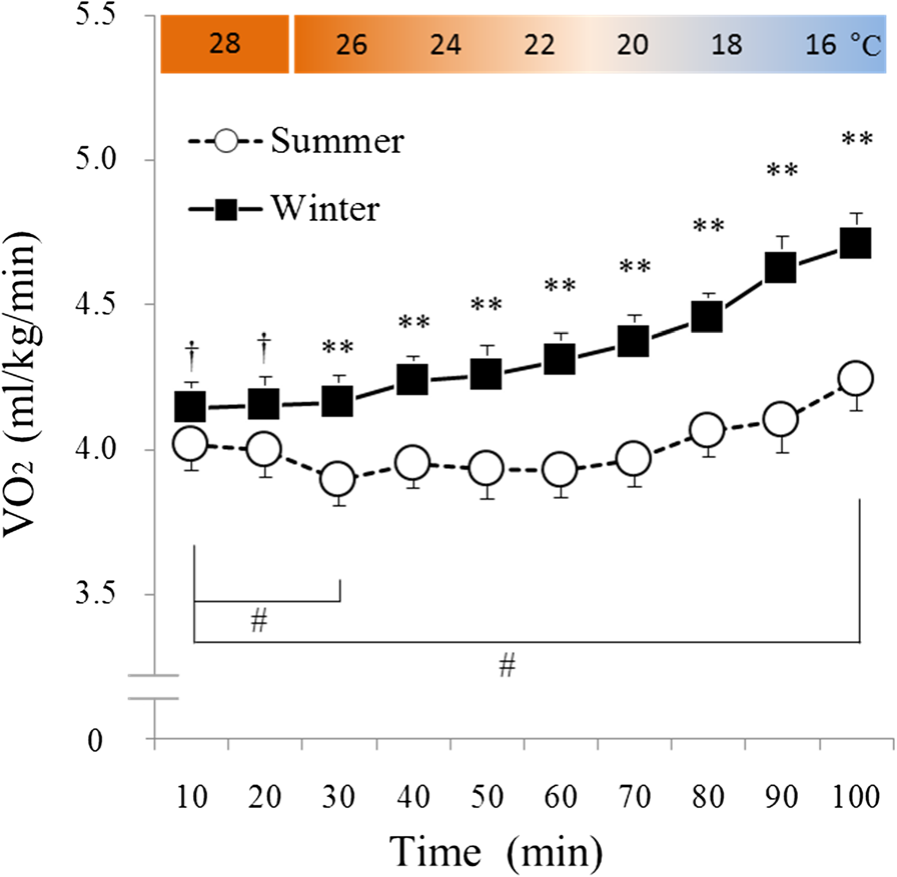
**Changes in oxygen intake (VO**_**2**_ **) during cold exposure.** White circles with the dotted line indicate summer data (*n* = 17, mean ± S.E), and black squares with the solid line indicate winter data (*n* = 17, mean ± S.E). In winter, VO_2_ tended to be greater during thermoneutral baseline and was significantly greater in the period ranging from 30 to 100 min during cold exposure than it was during those same periods in summer. In winter, VO_2_ was significantly greater after 40 min of cold exposure than it was during the first 10 min. In summer, VO_2_ was significantly lower after 30 min of cold exposure and tended to be greater after 100 min of cold exposure than it was during the first 10 min. ^†^ *P* < 0.1, **P < 0.01 when summer and winter values were compared. ^*#*^ *P* < 0.05 when summer values were compared with the other summer values.

The effects of season (*F* (1, 16) = 23.86, *P* < 0.001) and time (*F* (9, 144) = 15.54, *P* < 0.001) were significant with respect to oxygen intake (Figure 2). A significant interaction between season and time was also observed (*F* (9, 144) = 5.79, *P* < 0.001). In a *post hoc* test conducted using winter data, VO_2_ tended to be greater during thermoneutral baseline conditions and was significantly greater in the period ranging from 30 to 100 min during cold exposure than it was during the same period in summer. In summer, VO_2_ was significantly lower during the first 30 min of cold exposure compared with the thermoneutral baseline and tended to be greater after 100 min of cold exposure than the thermoneutral baseline.

### Change in electromyogram

**Figure 3:**
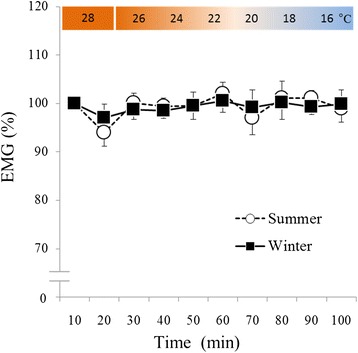
**Change in electromyogram.** White circles with the dotted line indicate summer data (*n* = 17, mean ± S.E), and black squares with the solid line indicate the winter data (*n* = 17, mean ± S.E).

The data are based on changes during the first 10 min. There were no significant effects of season and time, and there was no significant interaction between season and time (Figure 3).

### Respiratory exchange ratio

**Figure 4:**
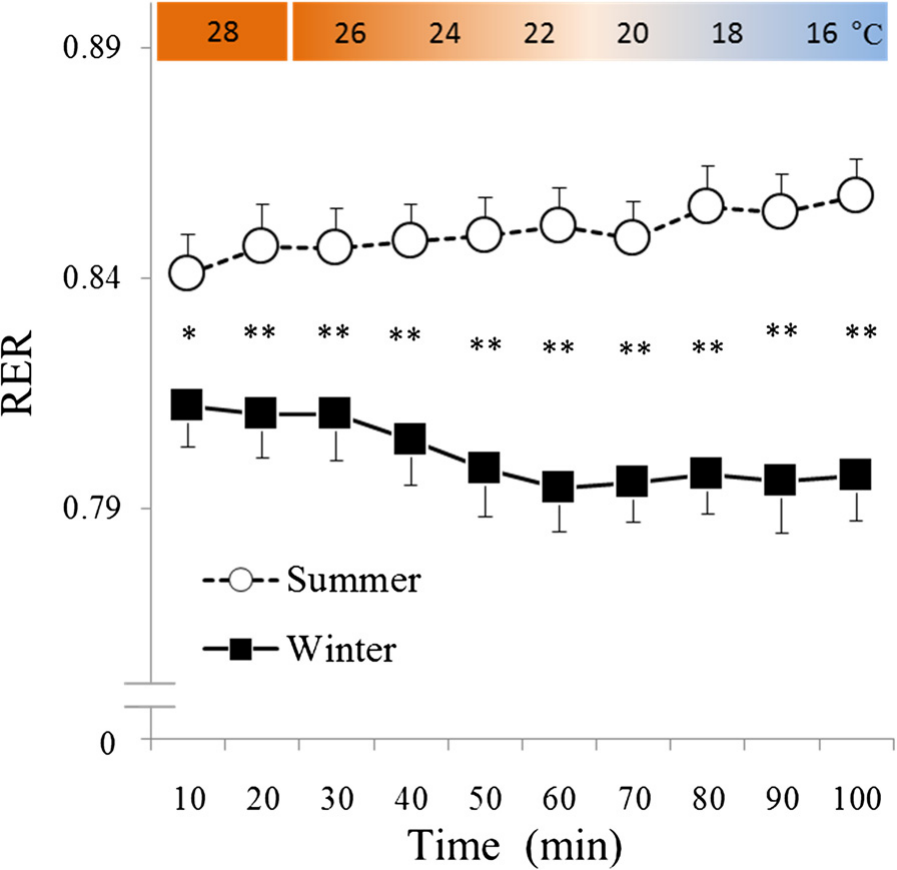
**Changes in respiratory exchange ratio (RER).** White circles with the dotted line indicate summer data (*n* = 17, mean ± S.E), and black squares with the solid line indicate winter data (*n* = 17, mean ± S.E). In winter, RER was significantly greater over the course of the experiment than it was in summer. In addition, RER was significantly lower during cold exposure in winter than it was during that same period in summer. *P < 0.05, **P < 0.01 when summer and winter values were compared.

The effect of season (*F* (1, 16) = 19.77, *P* < 0.001) was significant with respect to RER (Figure 4). A significant interaction between season and time was also observed (*F* (9, 144) = 3.47, *P* < 0.001). In a *post hoc* test, RER was significantly lower over the course of the experiment in winter than it was in summer. In a *post hoc* test conducted using winter data, RER was significantly lower during periods of cold exposure.

### Correlation between ΔRER and ΔVO_2_ over the course of 100 min of cold exposure

**Figure 5:**
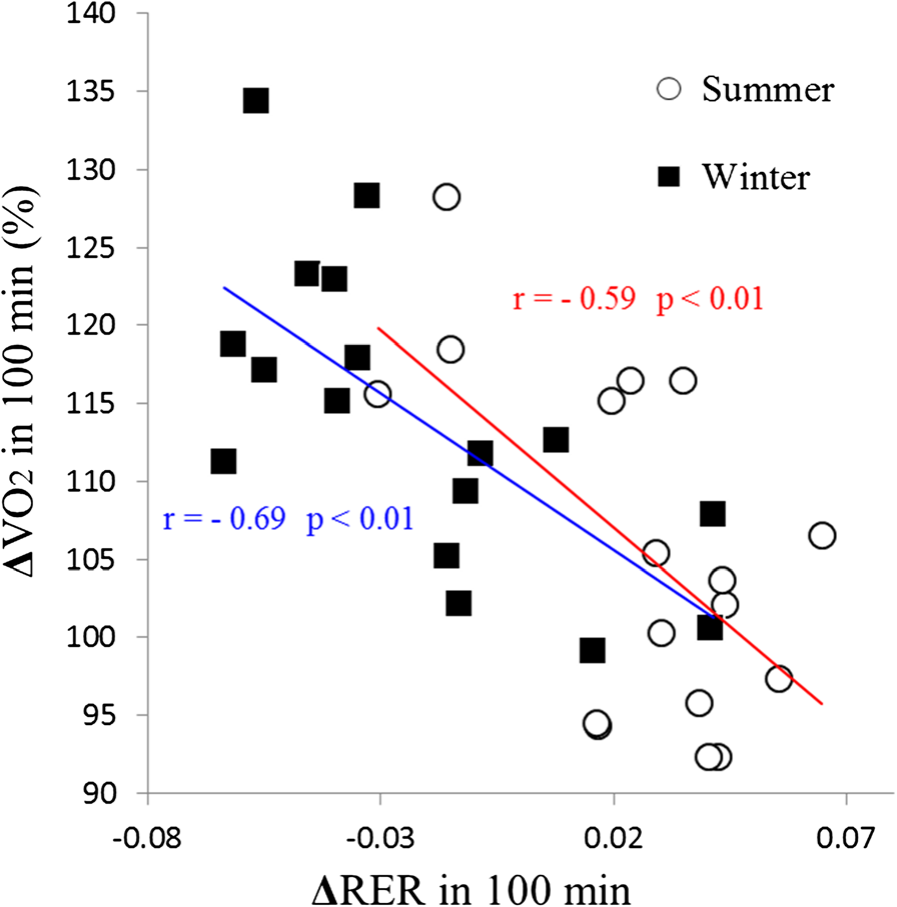
**Correlation between ΔRER and ΔVO**_**2**_ **over 100 min of cold exposure.** White circles indicate summer data (*n* = 17), and black squares indicate winter data (*n* = 17). ΔRER exhibited a significant negative relationship with ΔVO_2_ in both summer (*r* = −0.59) and winter (*r* = −0.69). The red line shows regression lines of ‘summer plots’ and blue line shows ‘winter plots’.

ΔRER was ‘subtract 10 min from 100 min’ data, and ΔVO_2_ were calculated using ‘ratio of 100 min from 10 min’ data (%) (Figure 5). ΔRER exhibited a significant negative relationship with ΔVO_2_ in both summer (*r* = −0.59, *P* < 0.01) and winter (*r* = −0.69, *P* < 0.01).

### Individual differences of ΔVO_2_ at 100 min in summer and winter

**Figure 6:**
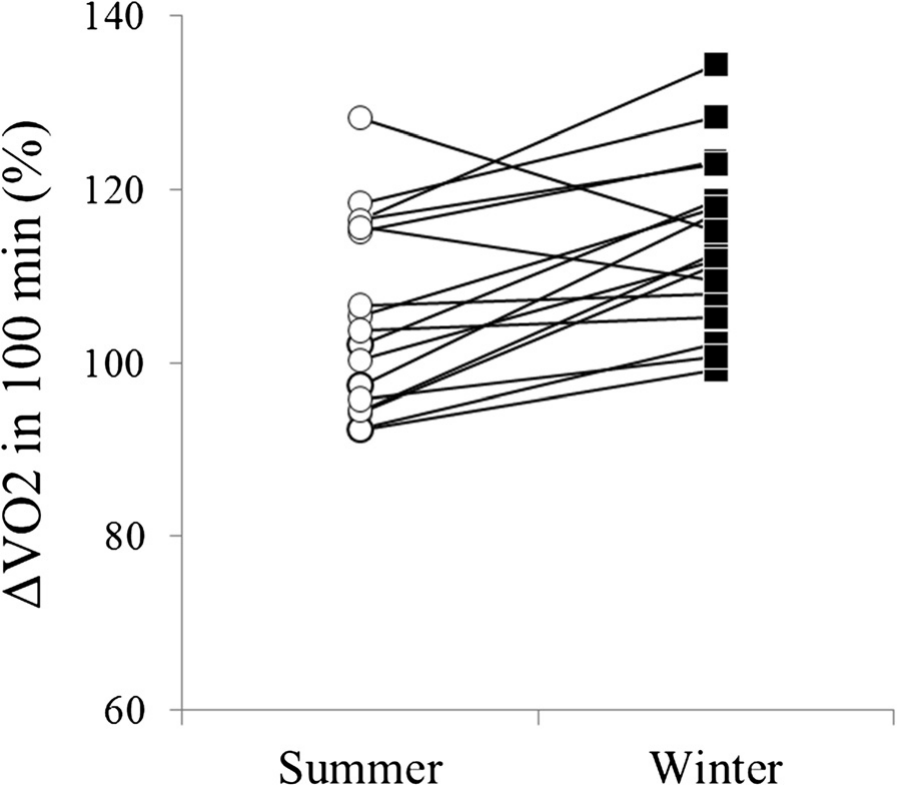
**Individual differences of ΔVO**_**2**_ **at 100 min in summer and winter.** White circles indicate the individual data of summer, and black squares indicate the individual data of winter.

Most of the participants showed greater increase in VO_2_ in winter than in summer, but some showed no seasonal difference or a greater increase in summer than in winter (Figure 6).

### Rectal temperature

**Figure 7:**
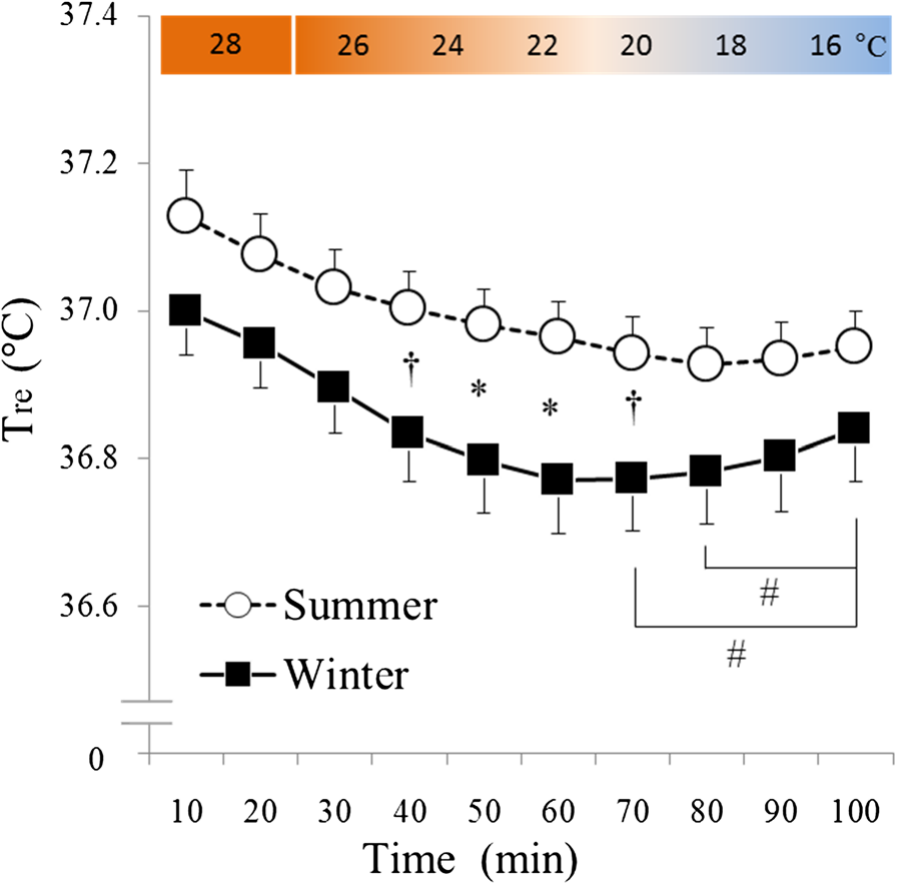
**Changes in rectal temperature.** White circles with the dotted line indicate summer data (*n* = 17, mean ± S.E), and black squares with the solid line indicate winter data (*n* = 17, mean ± S.E). T_re_ tended to be lower in the period between 40 and 70 min during cold exposure and was significantly lower in the period between 50 and 60 min during cold exposure in winter than it was during the same periods in summer. Furthermore, in winter, T_re_ was significantly greater at 100 min than it was in the period between 70 and 80 min during cold exposure. ^†^ *P* < 0.1, *P < 0.05 when summer and winter values were compared. ^*#*^ *P* < 0.05 when winter values were compared within the other winter values.

The effect of time (*F* (9, 144) = 29.17, *P* < 0.001) was significant with respect to T_re_ (Figure 7). A significant interaction between season and time was also observed (*F* (9, 144) = 2.45, *P* < 0.05). In a *post hoc* test, T_re_ tended to be lower in the period ranging from 40 to 70 min during cold exposure and was significantly lower in the period ranging from 50 to 60 min during cold exposure in winter than it was during the same period in summer. Furthermore, in winter, T_re_ was significantly higher at 100 min than it was between 70 and 80 min during cold exposure.

### Distal skin temperatures

The effects of season (*F* (1, 16) = 6.49, *P* < 0.05) and time (*F* (9, 144) = 2325.09, *P* < 0.001) were significant with respect to ${\overline{\mathrm{T}}}_{\mathrm{dist}}$ (Figure 7). A significant interaction between season and time was also observed (*F* (9, 144) = 31.41, *P* < 0.001). In a *post hoc* test, ${\overline{\mathrm{T}}}_{\mathrm{dist}}$ was significantly lower in the period ranging from 10 to 50 min during cold exposure.

## Discussion

In the present study, VO_2_ significantly and rapidly increased during winter (Figure 2) without shivering (Figure 3). In addition, RER was significantly lower during thermoneutral baseline conditions and periods of cold exposure in winter than in summer (Figure 4). However, in summer, VO_2_ was lowest at 30 min and highest at 100 min of cold exposure (Figure 2), and RER remained unchanged during cold exposure as compared to RER values recorded during thermoneutral baseline conditions (Figure 4). Although the heat source of NST remains unclear, brown adipose tissue (BAT) seems to account for the majority of heat generated by metabolizing free fatty acids [9,10] in this way. Previous studies have demonstrated seasonal variation in BAT activity [11-13]; with the majority of individuals having exhibited greater BAT activity levels in winter than in summer, and a minority of individuals having exhibited increased BAT activity during both seasons [11]. Similarly, in the present study, the majority of the participants exhibited increases in VO_2_ ranging from 20% to 30% in winter (Figure 6), while a minority of participants also exhibited increased VO_2_ during summer. Some individuals did not exhibit increased VO_2_ in either season (Figure 6). In addition, a significant correlation was observed between ΔVO_2_ and ΔRER (Figure 5), which indicated that RER was low, because increased fat metabolism (decreased RER) would result in greater VO_2_ in winter. This finding indicates that an individual with increased NST (ΔVO_2_) might be metabolizing more fat via BAT (decreased ΔRER), which supports inter-individual differences in NST intensity. These results suggested that NST might be affected by seasonal acclimatization or individual differences in BAT activity.

Basal metabolic rate (BMR) is responsible for obligatory NST in humans and tends to be greater in winter than in summer [14,15]. However, recent studies have indicated that air conditioners are capable of eliminating seasonal variation in BMR [16]. However, although the present study did not measure BMR, VO_2_ tended to be higher during thermoneutral conditions in winter than it did during the same periods in summer (Figure 2). In addition, some studies have reported NST generated from skeletal muscle [17,18]. Future studies should examine the relationship between NST of skeletal muscle and BMR in greater detail.

**Figure 8:**
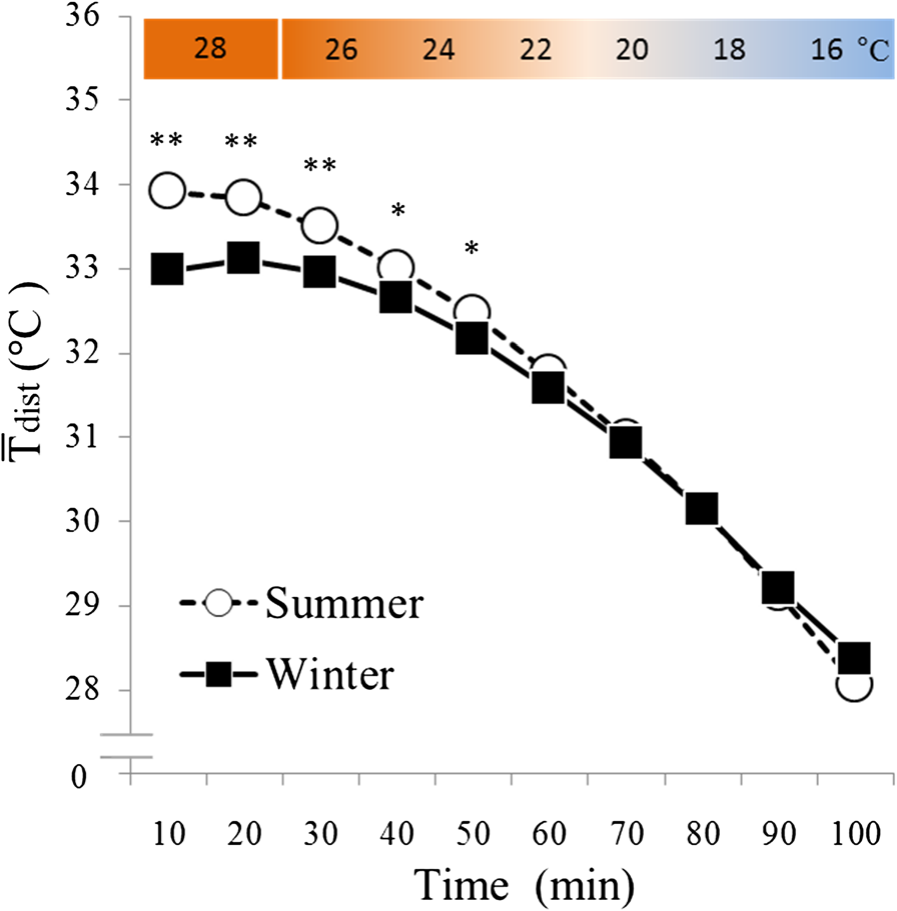
**Changes in distal skin temperatures.** White circles with a dotted line indicate summer data (*n* = 17, mean ± S.E), and black squares with a solid line indicate winter data (*n* = 17, mean ± S.E). *P < 0.05, **P < 0.01 when summer and winter values were compared. In winter, ${\overline{\mathrm{T}}}_{\mathrm{dist}}$ was significantly lower in the period between 10 and 50 min during cold exposure than it was during the same period in summer. *P < 0.05, **P < 0.01 when summer and winter values were compared.

T_re_ was lower during periods of cold exposure in winter than it was during the same periods in summer (Figure 7). This result was similar to those of previous studies [3,4]. Previous studies have also reported that, to prevent heat loss, skin blood flow was reduced in winter [19], resulting in lower distal skin temperatures, as in the present study (Figure 8). These results indicated that significant vasoconstriction did occur, especially in the foot in winter. Based on the observations noted above, it was suggested that the prevention of heat loss due to vasoconstriction in the foot occurs in response to mild cold exposure in winter.

The limitations of the present study include the fact that it did not directly measure BAT activity. It is necessary to measure amounts of BAT by positron emission tomography. In addition, all participants in the study had normal BMI values. Physiological data should be obtained from subjects with BMI values above and below the normal range. Moreover, behavioral or eating habits need to be studied to achieve an understanding of individual variation, because such habits may influence physiological data. Although cessation of eating/drinking was almost the same in summer and winter, the time used (2 h) was not sufficient to constitute fasting. More detailed data on the effects of fasting time are needed. Future studies should also examine possible genetic factors contributing to individual differences in NST or BAT activity, such as gene polymorphisms [20-22], to better understand the nature of the population level and individual variation in the trait.

## Conclusion

Increase of VO_2_ without shivering indicated an increase of NST, and a decrease of RER depends on the metabolization of fat in winter. These results suggested that NST activity was activated by seasonal acclimatization, and individual variation of NST depends on individual variation of fat metabolism.

## Abbreviations

BAT: Brown adipose tissue
BMI: Body mass index
BSA: Body surface area
NST: Non-shivering thermogenesis
RER: Respiratory exchange ratio
ST: Shivering thermogenesis
VCO_2_: Carbon dioxide output
VO_2_: Oxygen intake
